# Baicalein Attenuates Disuse-Driven Skeletal Senescence in Association with Gut Microbiota Modulation

**DOI:** 10.3390/molecules31183336

**Published:** 2026-09-20

**Authors:** Xin Zhao, Tingting Ren, Wei Bai, Hong Wang, Siddiq Ur Rahman, Xiaoni Deng, Kang Ru, Genyang Zhang, Wenjuan Zhang, Airong Qian

**Affiliations:** 1 Laboratory for Bone Metabolism, Xi’an Key Laboratory of Special Medicine and Health Engineering, Key Laboratory for Space Biosciences and Biotechnology, Research Center for Special Medicine and Health Systems Engineering, NPU-UAB Joint Laboratory for Bone Metabolism, School of Life Sciences, Northwestern Polytechnical University, Xi’an 710072, China; 2 School of Pharmacy, Shaanxi University of International Trade & Commerce, Xi’an 712046, China; 3 Research Center for Toxicological and Biological Effects, Institute for Hygiene of Ordnance Industry, Xi’an 710065, China; 4 Department of Computer Science and Bioinformatics, Khushal Khan Khattak University Karak, Karak 27200, Khyber Pakhtunkhwa, Pakistan; 5 Shenzhen Research Institute, Northwestern Polytechnical University, Shenzhen 518057, China

**Keywords:** baicalein, skeletal senescence, mechanical unloading, inflammatory response, gut microbiota

## Abstract

The progression of skeletal senescence under mechanical unloading conditions elevates fracture risk, emerging as a critical health challenge for the general population and astronauts during spaceflight. Nevertheless, current therapeutic strategies demonstrate persistent limitations in efficacy and safety, with unresolved challenges in long-term sustainability. Baicalein, a natural flavonoid compound, exhibits favorable anti-aging and anti-inflammatory effects, while its therapeutic potential and underlying mechanism in bone disorders remain unknown. In this study, a hind limb unloading (HLU) rat model was employed as a disuse simulation, after which the HLU rats received baicalein treatment by gavage at 30 mg·kg^−1^·day^−1^ for 4 weeks. Our results show that baicalein mitigates disuse-driven skeletal aging by ameliorating trabecular microstructure (Tb. N increased by 38.04%), accelerating bone mineral apposition rate (MAR increased by 42.76%), increasing bone mineral density (1.20-fold vs. model group), improving biomechanical strength (ultimate load +31.64%) and downregulating senescence-associated markers p16, p21, p53 in tibial tissue. Additionally, baicalein alleviated the unloading-induced high bone turnover, as evidenced by reducing serum levels of both osteogenic biomarkers ALP, PINP, BGP and osteoclastic biomarkers TRACP 5b, RANKL, NTX. Moreover, baicalein suppressed the inflammatory response caused by mechanical unloading, attributed to the restriction of pro-inflammatory factors TNF-α, IL-6, IL-8, IFN-γ and the promotion of anti-inflammatory cytokines IL-4, IL-10. Importantly, baicalein ameliorated the unloading-induced gut microbiota disorder through the diminished abundance of Proteobacteria and elevated abundance of Actinobacteria and Firmicutes, which were negatively associated with inflammatory response. Overall, our study provides evidence that baicalein inhibits the inflammatory response through the regulation of gut microbiota balance, which plays a role in preserving bone homeostasis.

## 1. Introduction

Disuse or immobilization weakens bone strength and structural integrity due to prolonged lack of mechanical loading, disrupting bone metabolism and triggering skeletal aging and degeneration [1,2]. It is currently a common condition in bedridden elderly patients and one of the hazardous diseases hindering long-term spaceflight missions for astronauts, severely decreasing their quality of life and increasing financial burden [3,4]. To alleviate this, pharmaceutical treatments, exercise training, mechanical stimulation, food supplementation have been developed to prevent further skeletal senescence and bone decline from occurring as a result of disuse [5,6]. Nevertheless, current therapeutics have limited and defective effects in mitigating initial disuse-driven skeletal senescence, and are often accompanied by significant side effects, including gastrointestinal disorders and adverse cardiovascular effects, which to some extent restrict their long-term usage and efficacy [7,8,9]. Accordingly, it is crucial to identify alternative therapeutic strategies to prevent disuse-associated skeletal senescence and mitigate the increased risk of fracture.

The natural active compounds derived from the homology of medicine and food, with low adverse effects and high safety, perform a pivotal role in preserving skeletal homeostasis and promoting intestinal health [10,11]. Baicalein, a bioactive flavonoid compound derived from *Scutellaria baicalensis*, has garnered attention for its diverse pharmacological properties, including anti-inflammatory, anti-aging, antioxidant, immunostimulating and microbiota-modulating activities [12,13,14]. Notably, emerging evidence suggests that inflammation acts as a critical regulator in skeletal aging, as gravitational mechanical unloading triggers systemic and local inflammatory responses marked by elevated pro-inflammatory cytokines, tumor necrosis factor-alpha (TNF-α) and interleukin-6 (IL-6), which exacerbate osteoclastogenesis and bone resorption [15]. Concurrently, the crosstalk between bone metabolism and the intestinal microbiome has been implicated in affecting bone homeostasis by regulating the immune and inflammatory state of the gastrointestinal tract [16,17]. A study suggested that the ratio of Firmicutes to Bacteroidetes (F/B ratio) in the intestinal microbiome of osteoporotic rats was dramatically increased, implying significant differences in microbial composition between osteoporosis and normal rodents [18,19]. Intriguingly, baicalein’s dual capacity to suppress inflammatory pathways and restore microbial homeostasis positions it as a promising candidate for combating disuse-mediated skeletal aging and deterioration. However, its protective effects and underlying mechanism on disuse-driven skeletal senescence, particularly its interplay with inflammation and gut microbiota, remain unexplored. We hypothesized that baicalein attenuates HLU-induced bone loss by remodeling the gut microbiota balance and suppressing intestinal inflammation.

In this study, the hindlimb unloading (HLU) model in rats was established to simulate an unloading condition, and the role of baicalein in ameliorating disuse-driven skeletal senescence and degradation was also investigated. Specifically, various indicators were examined and analyzed, including BMD, bone microstructure, biomechanical properties, bone formation rate, skeletal senescence markers, bone turnover biomarkers and inflammatory factors in serum. In addition, 16S rRNA high-throughput sequencing of fecal samples was integrated to elucidate the osteo-protective mechanism of baicalein. This study provides valuable insights into the potential protective effects and underlying mechanisms of baicalein against disuse-associated skeletal senescence. These findings may contribute to the future development of baicalein-based therapeutic strategies for preventing or mitigating disuse-induced skeletal deterioration.

## 2. Results

### 2.1. Baicalein Administration Exhibits No Adverse Effects on Rats

**Figure 1:**
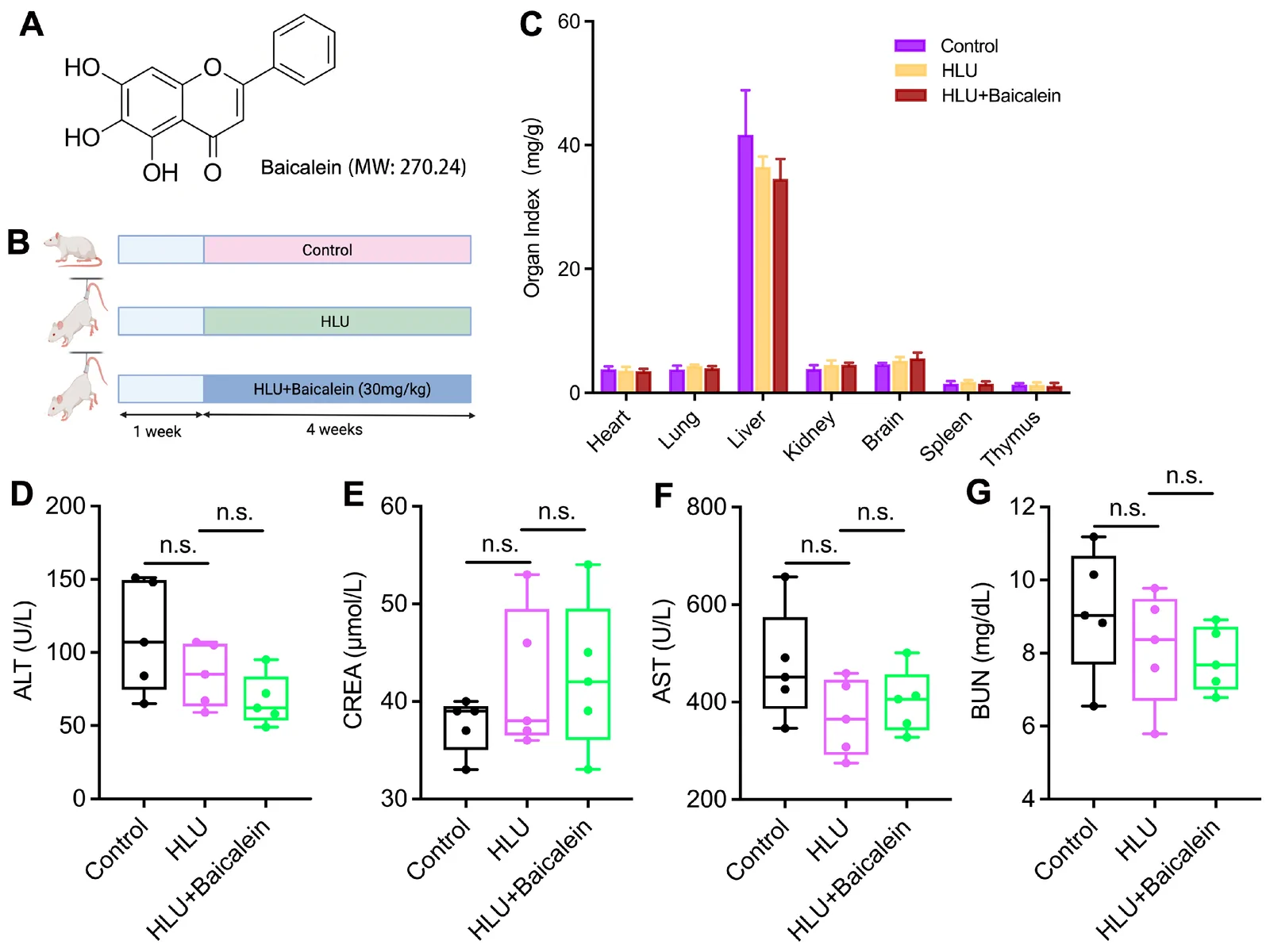
Effect of baicalein on the safety of experimental rats. (**A**) The chemical structure of baicalein. (**B**) Animal experimental design with three groups. (**C**) The organ index. (**D**–**G**) The levels of ALT, CREA, AST, BUN in serum. Data are shown as the mean ± SD; *n* = 6/group (n.s., not significant).

To investigate the therapeutic potential of baicalein against disuse-induced osteopenia and skeletal aging, the hindlimb unloading rat model was employed (Figure 1A,B). A comprehensive safety assessment was conducted to examine whether baicalein administration has potential adverse effects on rats, including organ indices of major organs and hepatorenal toxicity. There were no statistically significant differences in organ indices (heart, liver, spleen, lungs, brain, thymus and kidneys) across control, HLU, and HLU + Baicalein groups (*p* > 0.05, Figure 1C). The serum biochemical analyses of hepatic and renal function biomarkers ALT, AST, BUN, and CREA displayed the absence of marked alterations in levels across the three groups (*p* > 0.05, Figure 1D–G), demonstrating preserved hepatorenal homeostasis and a favorable biosafety profile of this intervention. Collectively, these findings demonstrate that baicalein exhibits no detectable adverse effects on organ integrity and systemic toxicity in rats, supporting its potential as a safe therapeutic candidate for mitigating disuse-associated skeletal disorder.

### 2.2. Baicalein Ameliorated Bone Microarchitecture in HLU-Exposed Rats

**Figure 2:**
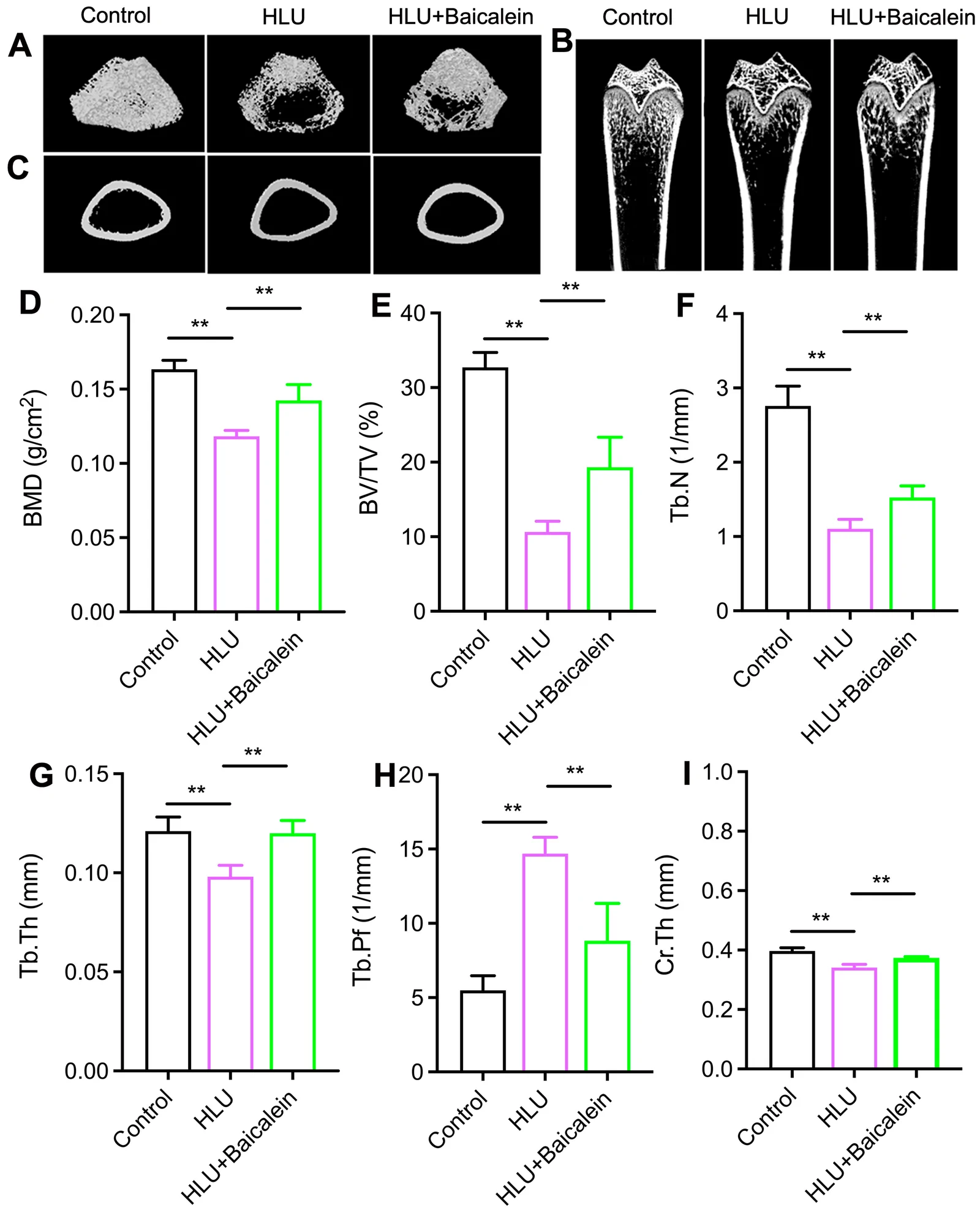
Effect of baicalein on bone microarchitecture. (**A**,**C**) Representative 3D images of trabecular and cortical bone of distal femurs. (**B**) Representative tomographic images of trabecular bone of distal femurs. (**D**–**I**) Quantitative analysis of BMD, bone volume per tissue volume (BV/TV), trabecular number (Tb.N), trabecular thickness (Tb.Th), trabecular bone pattern factor (Tb.Pf), cortical bone thickness (Cr.Th). Data are shown as the mean ± SD; *n* = 5, 6/group (** *p* < 0.01).

To explore the role of baicalein on bone mass, micro-CT analysis was performed to assess trabecular and cortical bone microarchitecture in rat femurs. Representative three-dimensional (3D) images of trabecular bone and cortical bone, and representative tomographic images of trabecular bone, evidenced a substantial decline in the degree of bone trabecular junction in HLU rats, whereas baicalein supplementation effectively improved the microstructural damage to bone trabeculae (Figure 2A–C). Quantitatively, the HLU group exhibited significantly lower values in trabecular bone parameters BV/TV, Tb.Th, Tb.N, BMD and cortical bone thickness Cr.Th than in controls, along with a marked elevation in Tb.Pf (*p* < 0.01). However, baicalein supplementation improved bone mass by increasing microstructure indices of femur BV/TV, Tb.N, Tb.Th, Cr.Th, BMD and reducing Tb.Pf (*p* < 0.01, Figure 2D–I). Collectively, these results imply that baicalein administration effectively alleviated unloading-induced microstructure deterioration in the femurs.

### 2.3. Baicalein Improved Femoral Biomechanical Integrity in HLU-Exposed Rats

**Figure 3:**
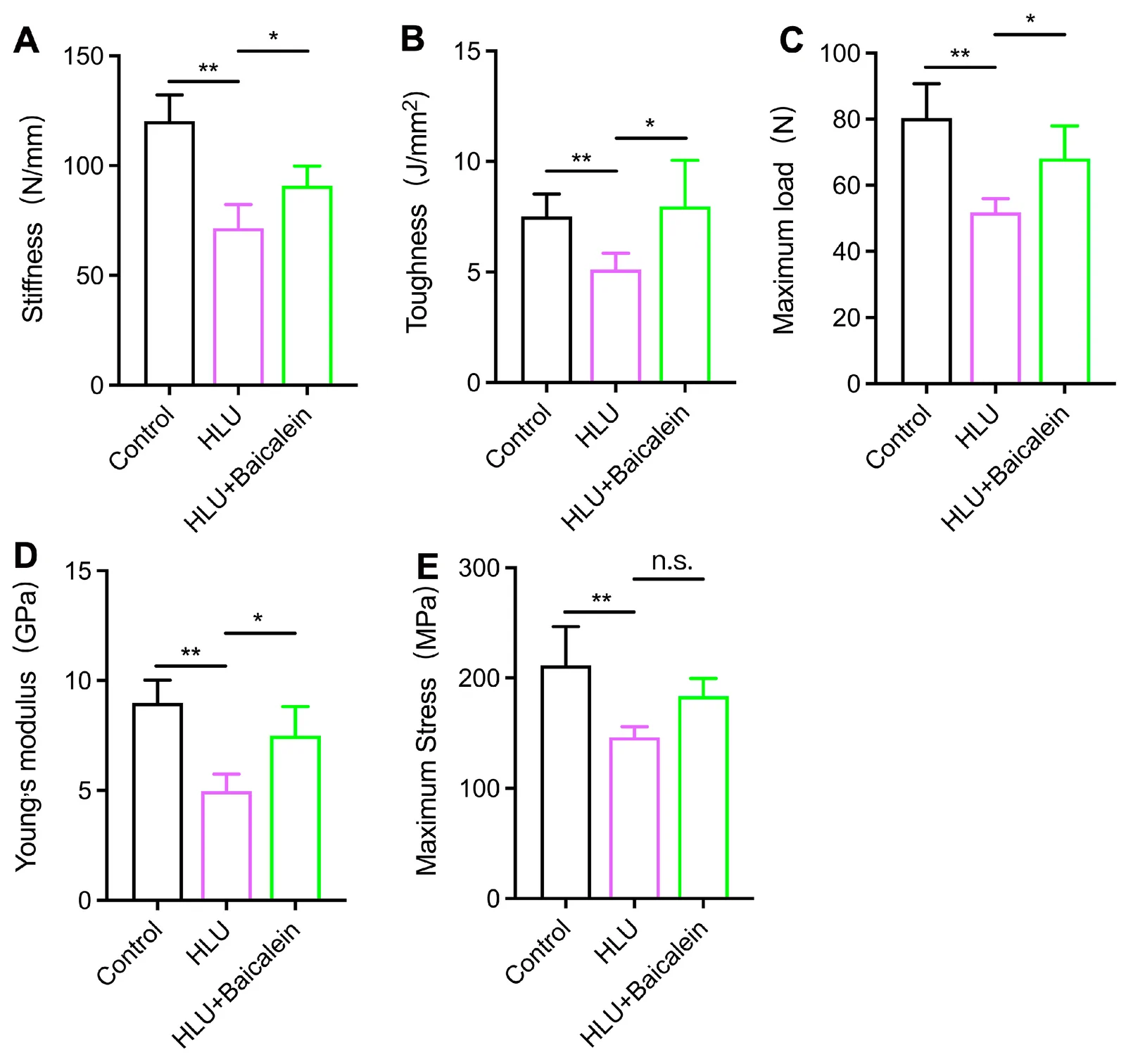
Effect of baicalein on the biomechanical properties of the femurs. This includes stiffness (**A**), toughness (**B**), maximum load (**C**), Young’s modulus (**D**), maximum stress (**E**) among three groups. Data are shown as the mean ± SD; *n* = 5, 6/group (* *p* < 0.05, ** *p* < 0.01, n.s., not significant).

To determine baicalein’s impact on HLU-induced alterations in biomechanical properties, three-point bending tests were systematically performed on femoral specimens. Biomechanical parameter analyses revealed significantly greater reductions in maximum stress, Young’s modulus, maximum load, stiffness, and toughness in the HLU group than in controls (*p* < 0.01, Figure 3A–E). Notably, baicalein administration partially ameliorated these HLU-induced deficits, with significant improvements observed in Young’s modulus, maximum load, stiffness, and toughness when compared with the HLU group (*p* < 0.05). Although no statistically significant change was detected in maximum stress between the HLU and HLU + Baicalein groups, baicalein had a certain enhancing effect on it (*p* > 0.05). In conclusion, while the increase in maximum stress did not reach statistical significance, baicalein significantly improved Young’s modulus, maximum load, stiffness, and toughness compared with the HLU group.

### 2.4. Baicalein Enhances Bone Matrix Mineralization in HLU-Exposed Rats

**Figure 4:**
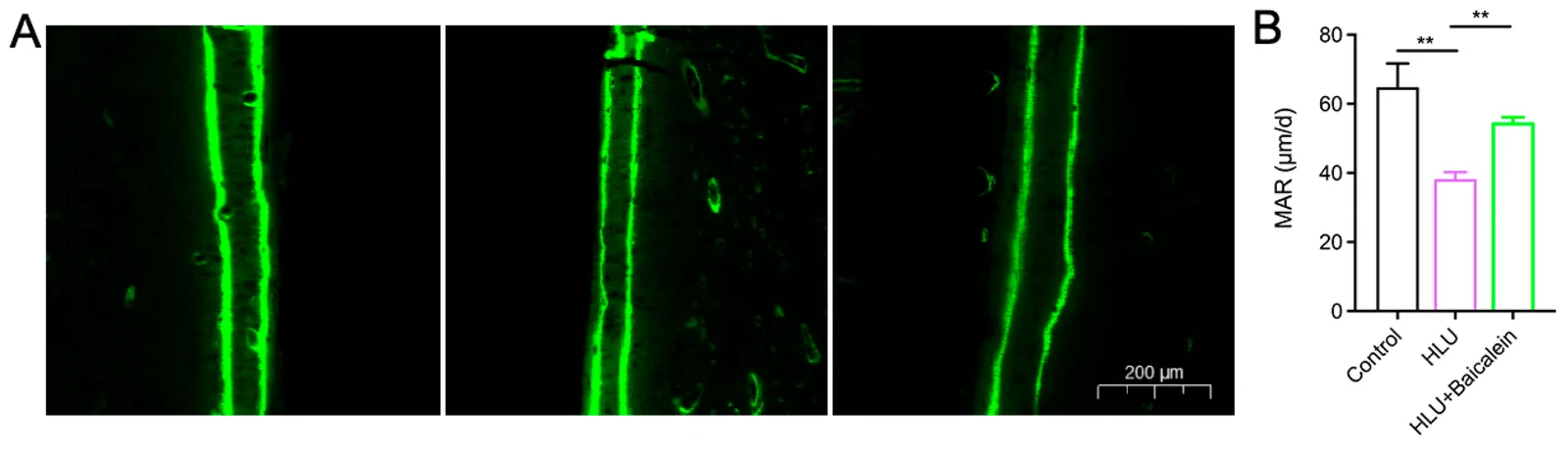
Effect of baicalein on the mineral apposition rate. (**A**) Representative fluorescence micrographs of trabecular bone samples showing green calcein labels within various groups (scale bar: 200 µm). (**B**) Quantitative analysis of the MAR. Data are shown as the mean ± SD; *n* = 3/group (** *p* < 0.01).

To evaluate the osteogenic potential of baicalein, the mineralization and deposition of the femurs in rats was examined. Representative fluorescent micrographs of trabecular bone sections within each group are shown in Figure 4A, where the distance between the fluorescent lines could reflect the deposition of bone calcification within the interval. The quantitative analyses of mineral apposition rate (MAR) are shown in Figure 4B. Dynamic histomorphometry revealed markedly slower calcification in HLU specimens compared with controls, with a reduction of 47.09% (*p* < 0.01). However, baicalein administration significantly increased the mineral apposition rate compared with the HLU group, with an increase of 42.76% (*p* < 0.01). Given the above results, baicalein was shown to mitigate bone deterioration in rats induced by tail suspension.

### 2.5. Baicalein Restored Bone Turnover Homeostasis in HLU-Exposed Rats

**Figure 5:**
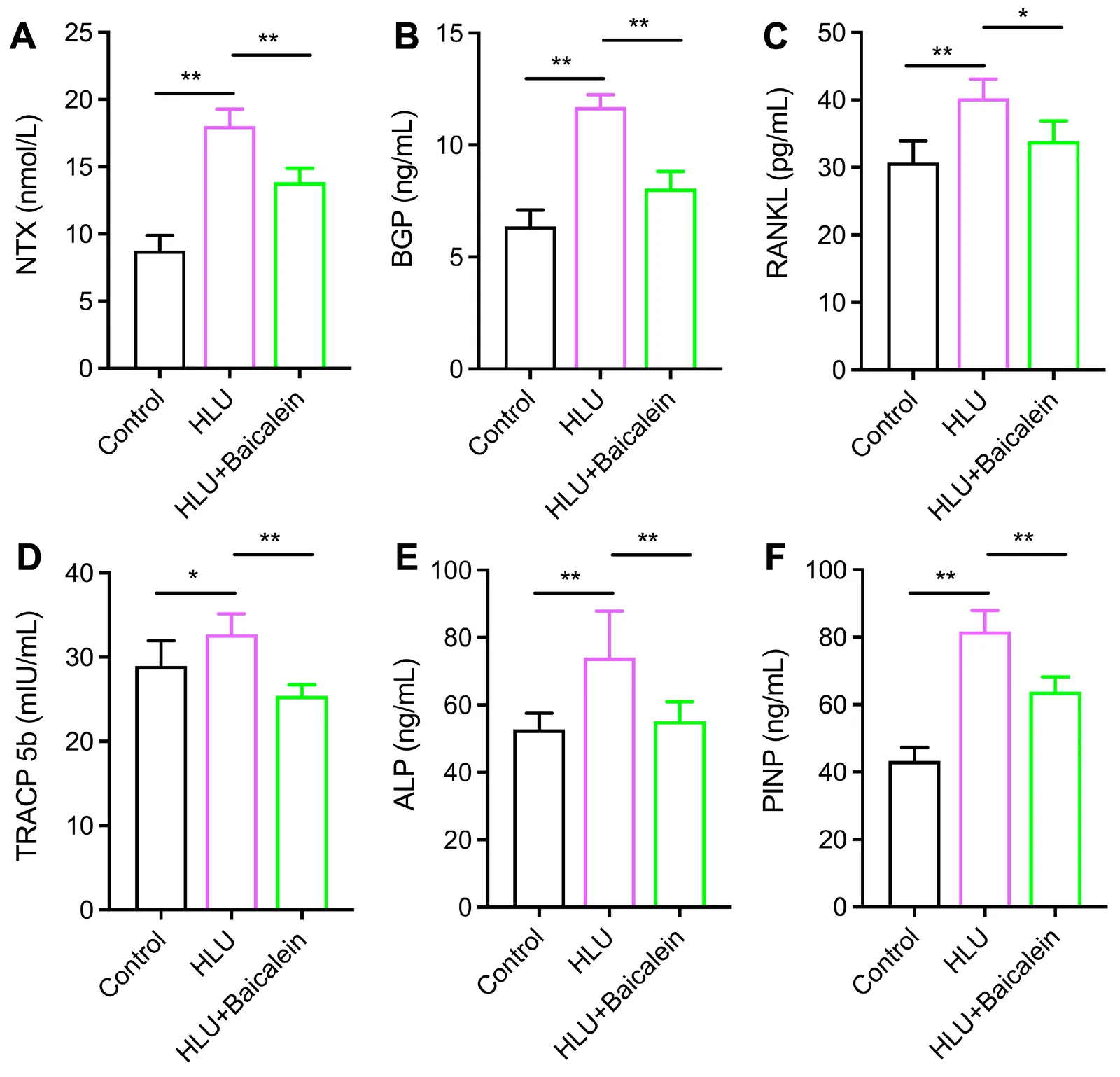
Effect of baicalein on the serum levels of bone turnover biomarkers as confirmed by ELISA analysis. This includes NTX (**A**), BGP (**B**), RANKL (**C**), TRACP 5b (**D**), ALP (**E**), and PINP (**F**) among three groups. Data are shown as the mean ± SD; *n* = 6/group (* *p* < 0.05, ** *p* < 0.01).

Bone turnover, a dynamic equilibrium between osteoblastic formation and osteoclastic resorption, is critical for skeletal integrity and metabolic homeostasis. Dysregulation of this process constitutes a hallmark of disuse osteopenia [20]. To elucidate the effect of baicalein on bone metabolism and its therapeutic efficacy in bone disorders, the serum concentrations of bone turnover biomarkers were determined. There was a significantly higher content of BGP, ALP, RANKL, TRACP 5b, PINP and NTX in the HLU group than in controls (Figure 5A–F, *p* < 0.01). Furthermore, baicalein treatment dramatically attenuated HLU-induced elevations in both osteogenic and osteoclastic serum biomarkers BGP, ALP, PINP, RANKL, TRACP 5b, NTX (*p* < 0.01). This implied that unloading enhanced bone turnover in rats and led to an imbalance in bone remodeling, while baicalein supplementation reduced these driving effects caused by tail suspension, indicating its important role in preserving bone homeostasis.

### 2.6. Baicalein Ameliorates Skeletal Senescence in HLU-Exposed Rats

**Figure 6:**
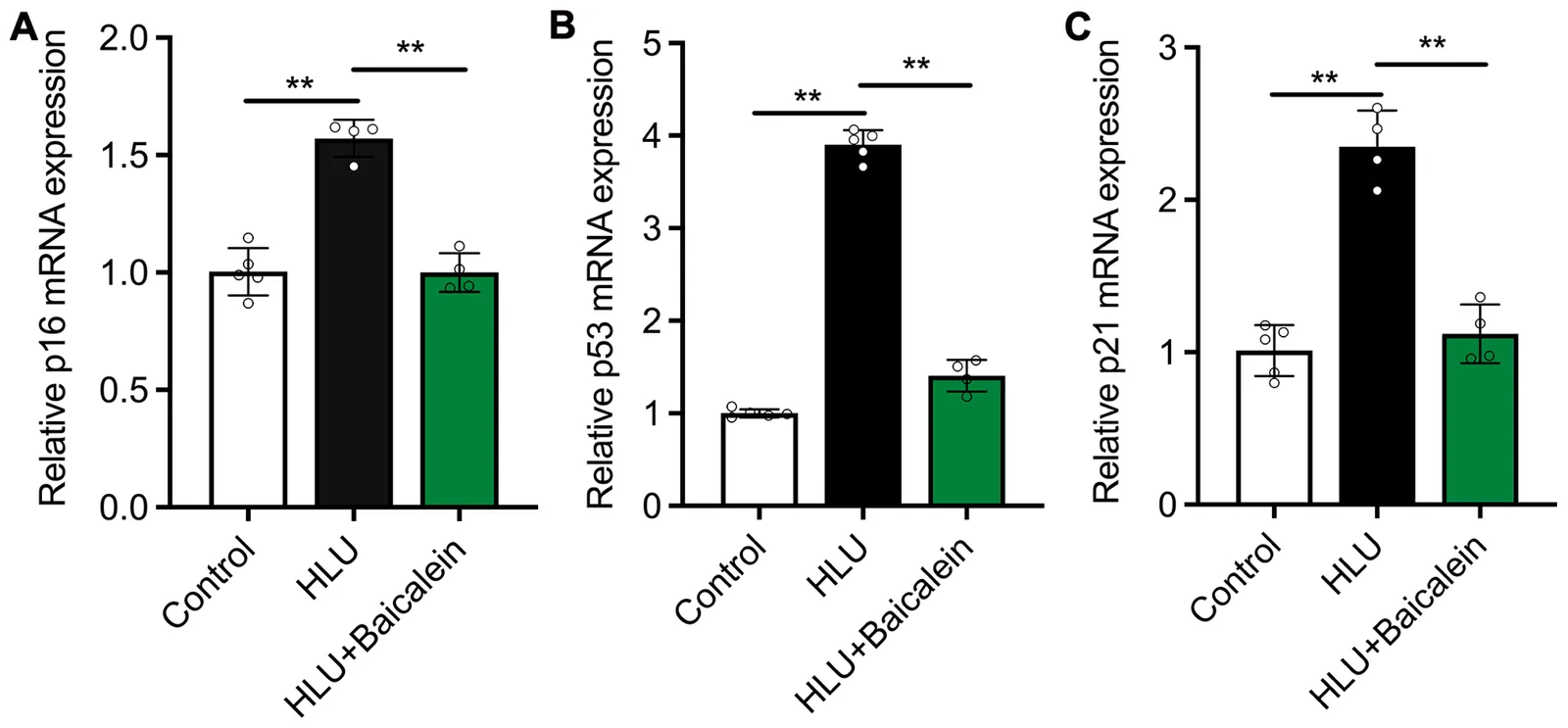
Effect of baicalein on the mRNA levels of senescence-associated genes in tibial tissue as validated by RT-PCR. This includes p16 (**A**), p53 (**B**) and p21 (**C**) across three groups. Data are shown as the mean ± SD; *n* = 4–6/group (** *p* < 0.01).

Senescence is a multifactorial process characterized by the gradual decline of tissue homeostasis and exacerbated by external stressors, such as mechanical unloading. The HLU group showed considerably higher mRNA expression of senescence-associated genes p16, p21, and p53 in rat tibial tissue compared with the control group, suggesting that disuse itself accelerated skeletal aging. However, baicalein supplementation markedly downregulated the mRNA levels of these senescence-related markers, demonstrating its dual role in counteracting senescence and alleviating bone degeneration (*p* < 0.01) (Figure 6A–C). The observations elucidate that baicalein exerts protective effects against both bone deterioration and senescence.

### 2.7. Baicalein Ameliorates Inflammation Response Induced by Disuse

**Figure 7:**
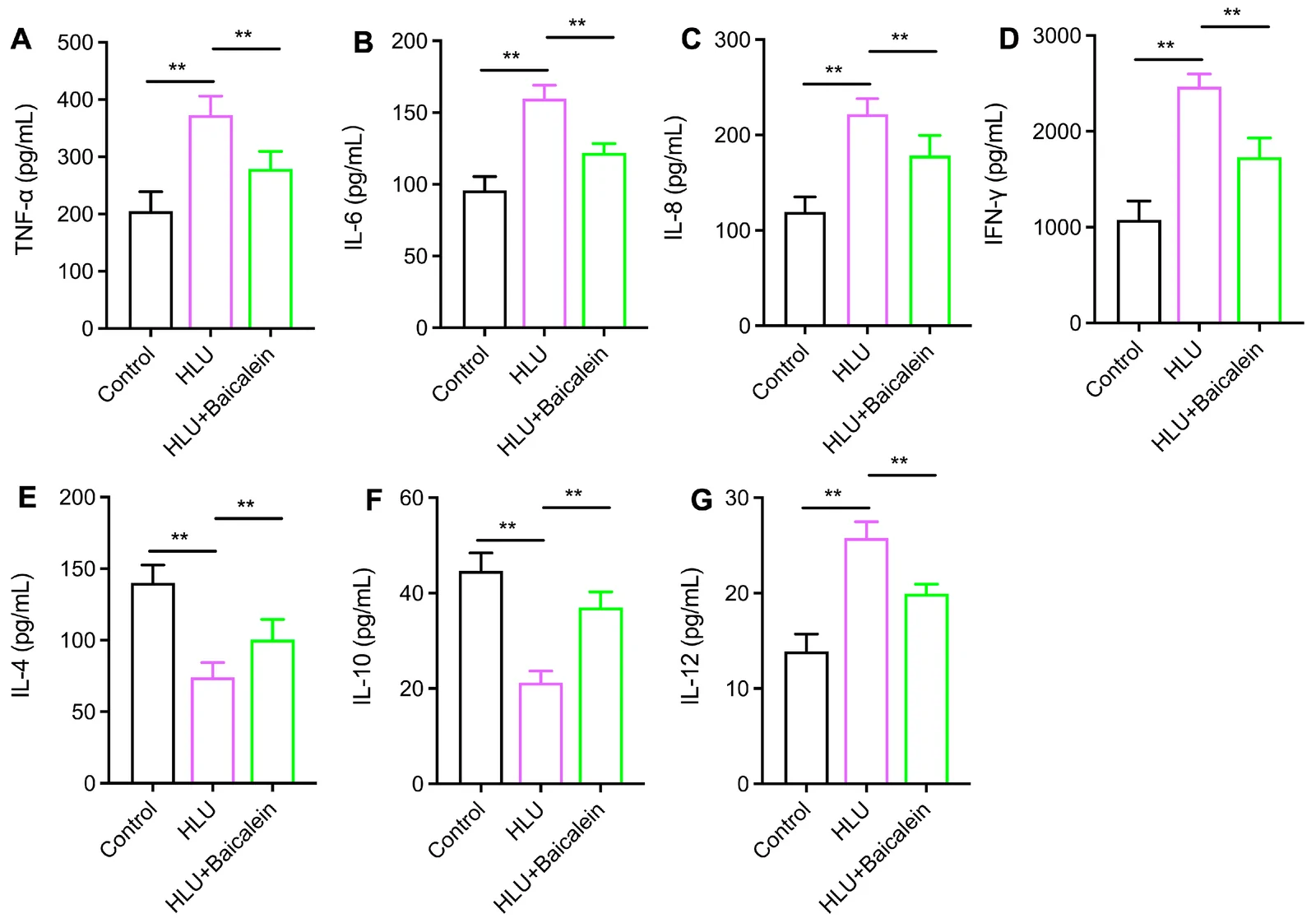
Effect of baicalein on the serum levels of inflammatory factors as investigated by ELISA analysis. This includes TNF-α (**A**), IL-6 (**B**), IL-8 (**C**), IFN-γ (**D**), IL-4 (**E**), IL-10 (**F**), and IL-12 (**G**) across three groups. Data are shown as the mean ± SD; *n* = 6/group (** *p* < 0.01).

Chronic inflammation caused by unloading is one of the main risk factors leading to skeletal senescence and degradation. To evaluate the effects of baicalein on the systemic inflammatory response induced by unloading, inflammatory factors in serum were investigated. We observed that the serum levels of TNF-α, IFN-γ, IL-8, IL-6 and IL-12 were increased in the HLU group, whereas baicalein supplementation noticeably decreased these levels (*p* < 0.01) (Figure 7A–G). The serum concentrations of IL-10 and IL-4 showed an obvious reduction in the HLU group, and this phenomenon was partially reversed by baicalein administration. These results reveal that baicalein supplementation attenuated the disuse-driven inflammatory response.

### 2.8. Baicalein Ameliorated the Composition of the Intestinal Flora in HLU-Induced Rats

**Figure 8:**
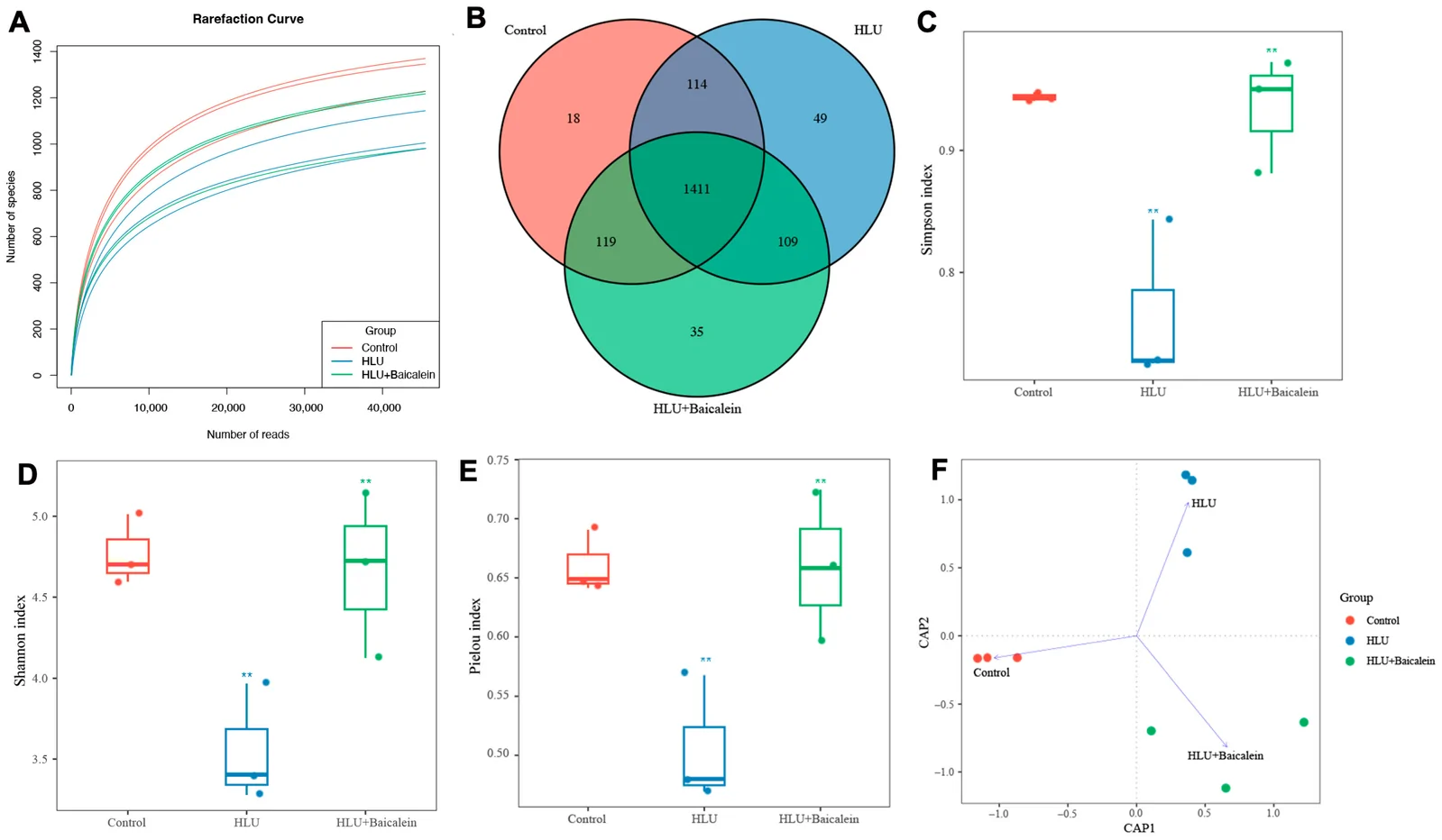
Effect of baicalein on the diversity and richness of intestinal flora. (**A**) Rarefaction curve of the sequenced samples. (**B**) Venn diagram of OTUs across three groups. (**C**) Simpson’s index. (**D**) Shannon’s index. (**E**) Pielou index. (**F**) CAP analysis displays the distribution and intergroup differences in fecal samples among three groups. Data are shown as mean ± SD, *n* = 3/group (** *p* < 0.01).

High-throughput sequencing was utilized to uncover significant alterations in the fecal microbiota. After the sample sequence reached 30,000 reads, the rarefaction curve flattened, signifying that the sequencing had proceeded to a point sufficient to capture the representative diversity of the microbial communities within the samples (Figure 8A). Consequently, it is reasonable to conclude that the sequencing data provided comprehensive microbial coverage, effectively reflecting the majority of species present in the specimens. The Venn diagram displays overlapping and distinct OTUs among three groups (Figure 8B). The observed OTU counts were 1622, 1683, and 1674 for the control, HLU, and HLU + Baicalein groups, respectively, while the total and common OTU counts across all groups were 1855 and 1411. Notably, the HLU + Baicalein group showed greater OTU overlap with controls than the HLU group (119 vs. 109), indicating partial microbiota restoration toward normal composition. Furthermore, a greater reduction in the α-diversity indicators (Simpson, Shannon and Pielou indices) was demonstrated in the HLU group than in controls, indicating a lower mean species diversity (*p* < 0.01). Importantly, these detrimental effects were mitigated by baicalein intervention (Figure 8C–E). The above findings collectively indicate baicalein has the potential to effectively restore the diminished richness and diversity of the intestinal flora induced by HLU.

**Figure 9:**
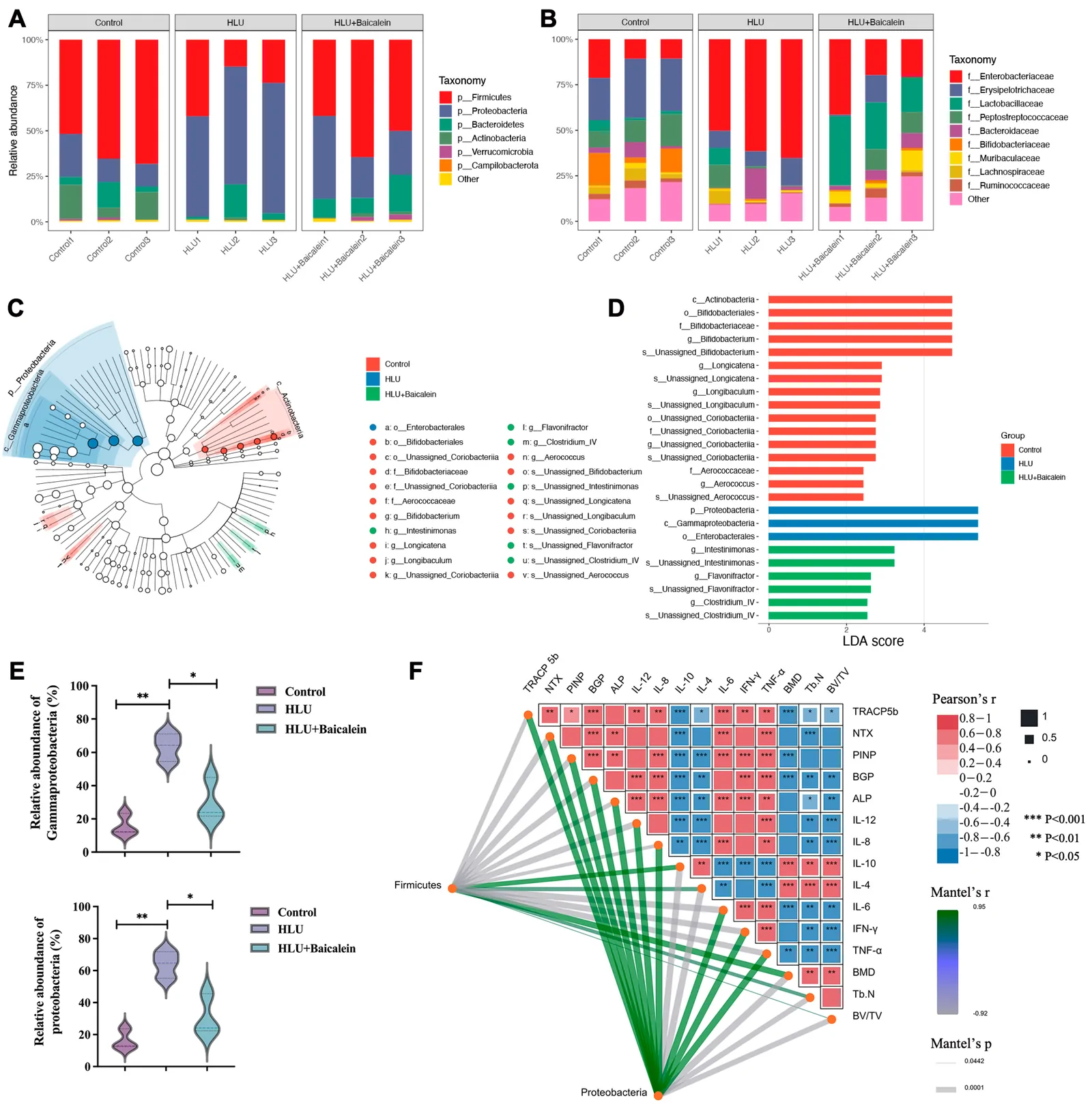
Effect of baicalein on the diversity and structure of intestinal flora. (**A**) Bacterial phylum-level classification of intestinal microbiota. (**B**) Bacterial family-level classification of intestinal microbiota. (**C**) Cladogram phylogenetic distribution of intestinal flora across three groups; (**D**) LEfSe analysis showing the differential microbiota among three groups. Taxa with an LDA score >2 are displayed. (**E**) Relative abundance of Gammaproteobacteria and Proteobacteria among three groups. (**F**) Relationship among the microbes and biochemical indices. Data are shown as mean ± SD, *n* = 3/group (* *p* < 0.05, ** *p* < 0.01, *** *p* < 0.001).

To gain a deeper insight into the variations in intestinal microbiota across various groups, we conducted an analysis focusing on the composition of flora at the phylum and family taxonomic levels. Microbiome profiling revealed Firmicutes and Bacteroidetes as the most predominant phyla, followed by Proteobacteria, Actinobacteria and Campilobacterota (Figure 9A). HLU led to a notable rise in Proteobacteria abundance when compared with the control group (16.35% vs. 63.88%, *p* < 0.01). Furthermore, a significant reduction in Actinobacteria (13.16% vs. 0.90%, *p* < 0.01) and Firmicutes abundance (61.76% vs. 26.81%, *p* < 0.01) was identified under the disuse condition, implying dysbiosis of the intestinal microbiota due to HLU exposure. Nevertheless, baicalein treatment appeared to decrease the relative abundance of Proteobacteria to 30.69%, while simultaneously reversing the relative abundance of Actinobacteria and Firmicutes to 1.43% and 52.14%, respectively (Figure 9A). Furthermore, microbial composition analysis identified Enterobacteriaceae, Erysipelotrichaceae and Lactobacillaceae as the core families, followed by Peptostreptococcaceae, Bacteroidaceae, Bifidobacteriaceae, and Muribaculaceae (Figure 9B). Importantly, the abundance of Enterobacteriaceae (58.99% vs. 27.27%, *p* < 0.01) and Bacteroidaceae (6.26% vs. 5.34%, *p* < 0.01) was obviously decreased after baicalein treatment, whilst the abundance of Muribaculaceae (0.85% vs. 6.41%, *p* < 0.01), Lactobacillaceae (3.10% vs. 27.47%, *p* < 0.01) and Ruminococcaceae were visibly increased (0.59% vs. 3.18%, *p* < 0.01). In conclusion, baicalein appeared to have resulted in the alteration of the microbiota structure at the phylum and family levels.

Additionally, the cladogram generated through Linear discriminant analysis Effect Size (LEfSe) provided enhanced insights into the intestinal flora composition across the three distinct groups, offering a clearer perspective on their differences. A total of 25 key intestinal taxa were identified, including 16 in the control group, 3 in the HLU group and 6 in the HLU + Baicalein group (Figure 9C,D). To be specific, noticeable microbial enrichment in the control group includes c_Actinobacteria, o_Bifidobacteriales, f_Bifidobacteriaceae, g_Bifidobacterium, s_Unassigned Bifidobacterium, g_Longicatena, Longibaculum. Regarding the HLU group, the differential microbes obtained were Proteobacteria, Gammaproteobacteria and Enterobacterales. Then, the abundance of the differential microbial communities Proteobacteria and Gammaproteobacteria caused by unloading showed an increase in the HLU group, while baicalein partially reversed these changes (Figure 9E). Additionally, noticeable microbial enrichment in the HLU + Baicalein group includes Intestinimonas and Flavonifractor genera. Taken together, these findings reveal baicalein is pivotal for regulating and normalizing the HLU-induced imbalance of gut microbiota.

To establish how gut microbiota relates to bone biomarkers, we conducted correlation analyses that revealed expected associations between microbial communities and parameters of skeletal and serum. Among them, Firmicutes demonstrated a positive correlation with bone mass BMD, Tb.N and serum inflammation IL-4 and IL-10 (*p* < 0.01, Figure 9F). Proteobacteria were negatively correlated with BMD, Tb.N, BV/TV and positively correlated with NTX, BGP, IL-4, IL-6 (*p* < 0.01). The observed correlation between gut flora and biological indicators reveals that intestinal dysbiosis may play a crucial role in driving bone destruction and skeletal senescence when exposed to HLU.

## 3. Discussion

Disuse-driven bone deterioration and skeletal senescence is caused by the weakening of mechanical stress stimulation on the skeleton, leading to the disruption of bone homeostasis and decline of bone mass, increasing the risk of osteoporosis and fractures [21]. Nevertheless, effective pharmacological interventions for this disorder remain lacking [22,23]. At present, some natural active compounds derived from homology of medicine and food have shown promising efficacy in ameliorating bone mass and mitigating osteoporotic progression [24]. Related studies have found that baicalein exhibits multiple pharmacological properties, including antioxidant, anti-inflammatory, and immune regulation [12,13]. Nevertheless, there is currently no evidence on the prevention and mechanism of unloading-induced bone deterioration and skeletal senescence. In this study, we conducted a systematic and comprehensive evaluation of baicalein’s potential to bone degeneration and skeletal senescence in vivo. Further, 16S rRNA high-throughput sequencing of fecal samples was utilized to clarify the underlying mechanism of baicalein on bone destruction and skeletal senescence at the gut–bone axis. This study provides evidence that baicalein, as a natural compound, may be potentially used in treating disuse-driven bone deterioration and senescence.

Hind limb unloading was recognized as an effective model for simulating force-unloading [25]. After tail suspension treatment, the reduction in BMD, bone mineral apposition rate, biomechanical properties, trabecular microarchitecture was demonstrated, indicating successful modeling and providing an important experimental basis for studying the prevention of disuse-induced skeleton deterioration and aging with baicalein. The administration of baicalein showed significant effects in improving bone quality, with a noticeable rise in BMD, BV/TV, Tb.Th, Tb.N, MAR and a remarkable reduction in Tb.Pf in rats. Bone turnover biomarkers serve to reflect both bone degeneration and regeneration processes, performing vital functions in sustaining bone homeostasis and metabolism [26]. This study revealed elevated concentrations of both osteogenic biomarkers ALP, PINP, BGP and osteoclastic biomarkers TRACP 5b, RANKL, NTX in the HLU group relative to controls, while baicalein restrained high bone turnover induced by mechanical unloading. In pathophysiological conditions, disruption of bone homeostasis leads to the development of skeletal senescence. This study revealed that unloading not only induced bone deterioration but also accelerated skeletal aging, as evidenced by the elevated expression of senescence-associated genes p16, p21 and p53 in the tibial tissue of the HLU group. Notably, baicalein treatment dramatically downregulated the mRNA levels of these senescence markers when compared with the HLU group, revealing its dual protective effects in ameliorating bone deterioration and counteracting senescence.

Healthy gut microbiota is characterized by a symbiosis of microbes with the host in the intestine, which provides various benefits to the host, including pathogen protection, nutrition, metabolism, and immunity [27,28]. To delve deeper into the mechanisms by which baicalein mitigates HLU-accelerated skeletal senescence, the alterations and characteristics of intestinal flora across three distinct groups were examined. In the present study, gravitational mechanical unloading substantially altered both the diversity and richness of gut microbiota, manifesting as reduced α-diversity and β-diversity profiles. Notably, HLU exposure led to a pronounced decline in microbial diversity, as investigated by significantly depressed values across all measured α-diversity indices. The coordinated improvement in Simpson, Pielou and Shannon indices following baicalein intervention confirms its efficacy in counteracting HLU-mediated α-diversity reduction. Additionally, CAP analysis of β-diversity revealed that HLU pronouncedly disrupted gut microbiota homeostasis, whereas baicalein exerted a crucial role in reversing the intestinal flora composition.

Our finding revealed that HLU disturbs the equilibrium of gut microbial communities, characterized by a marked expansion of Proteobacteria, a phylum often linked to inflammation and pathogenic states, and a decrease in probiotic taxa such as Firmicutes and Actinobacteria. These alterations corroborate previous studies demonstrating that gut dysbiosis, particularly an overabundance of Proteobacteria, may impair bone homeostasis by disrupting immune and exacerbating systemic inflammation [29,30]. Notably, baicalein administration partially restored microbial diversity and composition, as demonstrated by increased α-diversity indices and a shift in microbial profiles toward those observed in the control group. The reversal of Proteobacteria, Firmicutes and Actinobacteria abundance in the HLU + Baicalein group suggests that baicalein mitigates HLU-induced dysbiosis, potentially through its anti-inflammatory properties. The LEfSe analysis revealed distinct gut microbial signatures among the control, HLU, and HLU + Baicalein groups. Notably, the HLU group exhibited a marked enrichment of Proteobacteria, a phylum strongly associated with intestinal inflammation and barrier disruption [31,32]. Conversely, the control group was characterized by beneficial taxa, such as the gut commensal bacterium Bifidobacterium, which serves as a critical regulator in preserving bone homeostasis through host inflammatory responses. Supplementing with Bifidobacterium can noticeably diminish the serum levels of inflammatory cytokines and improve the microstructure of bone trabeculae in ovariectomized model mice [33]. Clinical evidence has also observed that the abundance of Bifidobacterium in the intestinal microbiota of osteoporosis patients is positively correlated with bone density [34]. Intriguingly, baicalein uniquely enriched Intestinimonas and Flavonifractor genera, which specialize in metabolizing flavonoids into bioactive derivatives [35,36]. This suggests a potential feedback loop: baicalein, a flavonoid, may select for bacteria that enhance its biotransformation, thereby amplifying its anti-inflammatory and osteoprotective effects. However, the precise mechanisms by which these microbial shifts influence bone health warrant further investigation, particularly through metabolomics. Importantly, correlation analyses underscored a strong association between specific microbial taxa and skeletal parameters. The positive correlation of Firmicutes with bone mass BMD, Tb.N and anti-inflammatory cytokines IL-4, IL-10 highlights its potential role in maintaining bone integrity, possibly via immune regulation and inflammatory response, which has been shown to enhance osteoblast activity [37]. Conversely, the negative correlation of Proteobacteria with bone metrics and its positive association with bone resorption markers NTX and pro-inflammatory cytokines IL-6 implicates this phylum in promoting osteoclastogenesis and inflammation-driven skeletal senescence. These findings are consistent with emerging evidence linking gut microbiota-derived signals to bone remodeling through the gut–bone axis [38,39].

## 4. Materials and Methods

### 4.1. HLU Rat Model Establishment and Baicalein Intervention

Twenty-four healthy male Sprague Dawley rats (body weight 200 ± 20 g) were housed under controlled environmental conditions (25 ± 3 °C; 12 h light/dark cycle). Following a 7-day acclimatization period, all rats were stochastically allocated into three experimental groups (*n* = 8 per group), including a control group, an HLU group, and an HLU treatment group with baicalein, treated for 4 weeks [40,41]. After randomization, no statistically significant differences in initial body weight were observed among the three groups, indicating comparable baseline body weights. During tail suspension, an elastic tape was attached to the tail of each rat in the HLU and HLU + Baicalein groups as recommended by Moreyholton and Globus [42]. The tape was connected to a clip secured to an overhead bar, positioning the rat at a 30° head-down tilt and lifting its hind limbs off the cage floor. Rats in the HLU + baicalein group were administered baicalein dissolved in 0.5% carboxymethylcellulose sodium (CMC-Na) by gavage at 30 mg·kg^−1^·d^−1^ daily, while the control and HLU groups received only an equivalent amount of 0.5% CMC-Na. Approval for the rat experiments was obtained from the Institutional Ethics Committee of Northwestern Polytechnical University (No. 202101111), and all procedures strictly followed the Guidelines for the Care and Use of Laboratory Animals.

### 4.2. Determination of the Organ Index

During the dissection of the rats, the main organs (heart, liver, spleen, lungs, brain, thymus and kidneys) were carefully harvested from each rat. These organs were gently washed with saline to remove any residual blood, then dried on filter paper to remove excess moisture. Following this, the organs were precisely weighed. A ratio of organ weight to body weight was subsequently computed as the organ index.

### 4.3. Bone Mineral Density (BMD) Assessment

The BMD of femurs was evaluated by dual-energy X-ray absorptiometry (DEXA) (Lunar Prodigy Advance DXA, GE healthcare, Chicago, IL, USA) [43]. The experimental rats underwent hindlimb unloading for 28 days. Anesthetic 10% chloral hydrate was administered via intraperitoneal injection, after which the anesthetized rats were carefully positioned on the DEXA scanner’s platform. A full-body scan of the rats was performed using the small-animal scanning mode while keeping them still. The femurs were then selected as the region of interest (ROI), and their BMD values were automatically analyzed using GE’s enCORE 14 software.

### 4.4. Micro-Computed Tomography

The bone microarchitecture of rat femurs was evaluated by micro-CT scanner (Skyscan 1276, Bruker microCT, Kontich, Belgium) [44]. The samples fixed in 4% paraformaldehyde were scanned along their longitudinal axis with the following parameters: a voltage of 70 kVp, a current of 114 μA, an image pixel size of 10 μm, and a layer spacing of 10 μm. Later, 3D femoral images were reconstructed and skeletal parameters were quantitatively analyzed by CTan 1.12 and CTvol 2.3.2 software. A 1-mm-thick trabecular bone region adjacent to the growth plate was designated as ROI for quantitative analysis. Cortical bone was excluded by manually contouring the inner cortical boundary on each slice, so that only the trabecular compartment was included. These parameters were included in the quantitative analysis, including bone volume per tissue volume (BV/TV), trabecular number (Tb.N), trabecular thickness (Tb.Th), trabecular bone pattern factor (Tb.Pf), and cortical bone thickness (Cr.Th).

### 4.5. Dynamic Bone Histomorphometry

Double calcein labeling was performed through subcutaneous administration (5 mg/kg) at two timepoints: 13 days and 3 days preceding necropsy. Following completion of the 4-week hindlimb unloading, the femurs were harvested and immediately fixed in 4% paraformaldehyde. Subsequent dehydration was performed through a graded ethanol series (60%-70%-80%-90%-95%) with immersion durations ranging from 24 to 72 h for each concentration. The dehydrated specimens were then transferred to embedding cassettes, infiltrated with methyl methacrylate resin under vacuum for 5 h, and polymerized in a 37 °C water bath. Polymerized bone blocks were sectioned into slices with a thickness of 10 μm using a hard tissue microtome (Leica SP1600, Nußloch, Germany). Digital histomorphometric analysis was conducted using a high-resolution slide scanning system with image acquisition performed through the manufacturer’s proprietary software (Pannoramic Viewer 2.3). Quantitative assessment of mineral apposition rate (MAR) was performed using CaseViewer 2.4 software following standardized protocols.

### 4.6. Three-Point Bending Mechanical Analysis

Femoral mechanical properties were assessed by a universal material tester via a standardized protocol (Intron company, Norwood, MA, USA) [10]. The sample was supported by two fixed points spaced 20 mm apart, with the load applied at the midpoint of the diaphysis. A downward force was exerted at a constant rate of 2 mm/min until fracture occurred. Throughout the test, the load and displacement data were continuously recorded by a connected computer. Mechanical properties were computed in MATLAB 9.8 (R2020a) based on the resulting load–displacement curve and fracture-site diameters (internal and external) measured via super-depth-of-field microscope on the fractured bone cross-section.

### 4.7. Bone Turnover Biomarkers and Inflammatory Cytokines in Serum

Serum was collected from blood by centrifuging at 1000× *g* at 4 °C for 10 min. Levels of bone turnover biomarkers and inflammatory factors were identified using enzyme-linked immunosorbent assay (ELISA) with available rat ELISA kits, following the manufacturer’s instructions. Specifically, absorbance values of both standards and serum samples were measured at the appropriate wavelength using a microplate reader. A standard curve was constructed from the standard absorbance readings, and the levels of bone turnover biomarkers BGP, ALP, PINP, RANKL, TRACP 5b, NTX and inflammatory factors TNF-α, IFN-γ, IL-8, IL-6, IL-10, IL-4, IL-12 in serum were interpolated from this curve. The ELISA kits for BGP(YX-151607R), ALP (YX-011216R), PINP (YX-160916R), RANKL (YX-180114R), TRACP 5b (YX-201801R), NTX (YX-142024R), TNF-α (YX-200906R), IFN-γ (YX-090614R), IL-8 (YX-091208R), IL-6 (YX-091206R), IL-10 (YX-091210R), IL-4 (YX-091204R), IL-12 (YX-091212R) were purchased from Shanghai Youxuan Biotechnology Co., Ltd. (Shanghai, China). The hepatorenal toxicity was detected by Beckman Coulter AU480 analyzer, including aspartate aminotransferase (AST), alanine aminotransferase (ALT), creatinine (CREA) and urea nitrogen (BUN).

### 4.8. RNA Isolation and Quantitative Real-Time PCR

Total RNA was extracted using TRIzol reagent (Invitrogen, Rockville, MD, USA) according to the manufacturer’s protocol. The concentration and purity of the extracted RNA were assessed by measuring the A260/A280 ratio via spectrophotometry. Following RNA quality assessment, 1 μg of total RNA was reverse-transcribed into cDNA using the PrimeScript RT reagent Kit (Invitrogen, Waltham, MA, USA). The sequences of the PCR primers are shown in Table 1. Quantitative real-time PCR was then performed to analyze gene expression levels, with relative mRNA expression normalized to GAPDH and calculated using the ΔΔCT method.

### 4.9. Fecal 16S rRNA Sequencing

For intestinal microbiota analysis, rat fecal samples were collected in sterile centrifuge tubes and immediately stored at −80 °C. The genomic DNA was extracted using Omega Mag-bind soil DNA kit (Omega M5636-02, Bienne, Switzerland) and DNA integrity was measured by 1.2% agarose gel electrophoresis. The specific primers were synthesized for the amplification of the V3-V4 regions of the 16S rDNA gene (F: ACTCCTACGGGAGGCAGCA; R: GGACTACHVGGGTWTCTAAT). The amplicon PCR was performed under the following conditions: initial denaturation at 98 °C for 2 min, followed by 25 cycles of denaturation at 98 °C for 15 s, annealing at 55 °C for 30 s, and extension at 72 °C for 30 s, with a final extension at 72 °C for 5 min, and then held at 10 °C. The amplification products were sequenced on an Illumina noveseq6000 platform (BioNovoGene Biotechnology Co., Ltd., Suzhou, China). Raw sequencing data underwent filtering of low-quality reads and merging of paired-end sequences, followed by operational taxonomic unit (OTU) clustering at 97% similarity and taxonomic annotation. On this basis, α-diversity and β-diversity indices of the microbiota were calculated. Among them, β-diversity was analyzed using Bray–Curtis dissimilarity with PERMANOVA. Bacterial community composition was analyzed for differences at both the phylum and family level. To identify variations in microbiota composition between groups, linear discriminant analysis combined with effect size (LEfSe) (LDA > 2, *p* < 0.05) was employed. The correlation analyses were performed using Spearman’s rank correlation with FDR correction.

### 4.10. Statistical Analyses

All data are presented as mean ± standard deviation (SD). Before statistical analysis, the data are analyzed using a normality test. Differences among various groups were evaluated by one-way analysis of variance (ANOVA) using GraphPad Prism 8.0 software, followed by Tukey’s HSD post hoc test. A value of *p* < 0.05 was regarded as statistically significant.

## 5. Conclusions

Collectively, our study shows that baicalein alleviates disuse-induced bone deterioration and skeletal senescence by ameliorating trabecular microstructure, accelerating bone mineral apposition rate, increasing bone mineral density, improving biomechanical strength, reducing bone turnover and downregulating senescence markers. Mechanistically, baicalein inhibits the inflammatory response, potentially in association with the enrichment of the Muribaculaceae, Lactobacillaceae and Ruminococcaceae and the reduction in the Enterobacteriaceae and Bacteroidaceae families, thereby contributing to the maintenance of bone health. Nevertheless, deeper mechanistic insights into baicalein for disuse-driven skeletal senescence warrant further investigation, with metabolomics, transcriptomics, intestinal barrier assessment and fecal transplantation to be deployed in subsequent studies.

## Figures and Tables

**Table 1:** The sequences of the PCR primers.

Primer	Sequence (5′ to 3′)	NCBI Gene ID
GAPDH	F: 5′-TGCACCACCAACTGCTTAG-3′	14433
	R: 5′-GGATGCAGGGATGATGTTC-3′	
p16	F: 5′-CCCGAACACTTTCGGTCGTA-3′	12578
	R: 5′-GCACCATAGGAGAGCAGGAG-3′	
p21	F: 5′-GTAGGACTTCGGGGTCTCCT-3′	12575
	R: 5′-AATGTCAAGGCTCTGGACGG-3′	
p53	F: 5′-TTGCCATTTTATGACTTTAGGG-3′	22059
	R: 5′-AACTGACCGGATAGGATTTCG-3′	

Note: F, forward primer; R, reverse primer.

## Data Availability

All data generated or analyzed during this study are included in this published article.
